# Supplementary material for: Machine learning the microscopic form of nematic order in twisted double-bilayer graphene
Source: Nat Commun. 2023 Aug 17;14:5012. doi: 10.1038/s41467-023-40684-1 (PMC10435506; doi:10.1038/s41467-023-40684-1)
Supplement: Supplementary file 1 — Supplementary Information [file 41467_2023_40684_MOESM1_ESM.pdf]

Supplementary Information for Machine learning the microscopic form of nematic order in twisted double-bilayer graphene

João Augusto Sobral,[1,*] Stefan Obernauer,[1] Simon Turkel,[2] Abhay Pasupathy,[2,3] and Mathias S. Scheurer[1,4]

[1] Institute for Theoretical Physics, University of Innsbruck, Innsbruck A-6020, Austria
[2] Department of Physics, Columbia University, New York, New York 10027, USA
[3] Condensed Matter Physics and Materials Science Division, Brookhaven National Laboratory, Upton, New York 11974, USA
[4] Institute for Theoretical Physics III, University of Stuttgart, 70550 Stuttgart, Germany

CONTENTS

Supplementary Note 1: Continuum model and LDOS maps 1

Supplementary Note 2: Including strain with fixed θ_ϵ, and variations of the ML architecture 2

Supplementary Note 3: Preprocessing of the experimental data and further implications 4

Supplementary Note 4: Details on training and robustness against pixel inhomogeneous disorder in $\mathcal{D}_{\omega_0}(\mathbf{r})$ maps 6

Supplementary Note 5: Applicability of the CNN to different models and moiré systems 8

SUPPLEMENTARY NOTE 1: CONTINUUM MODEL AND LDOS MAPS

Twisted double-bilayer graphene (TDBG) consists of two bilayer graphene stacks with primitive lattice vectors $\boldsymbol{a}_1 = a(1,0)$ and $\boldsymbol{a}_2 = a\left(1,\sqrt{3}\right)/2$, where $a \simeq 0.246$ nm is the lattice constant of graphene, and corresponding reciprocal lattice vectors $\boldsymbol{b}_j$, following from $\boldsymbol{a}_i \cdot \boldsymbol{b}_j = 2\pi\delta_{ij}$. After applying a twist angle θ between the Bernal stacks, these vectors are modified by the two-dimensional rotation matrix $R(\theta)$ as $\left(\boldsymbol{a}_i^l, \boldsymbol{b}_i^l\right) = R\left(\mp\theta/2\right)\left(\boldsymbol{a}_i, \boldsymbol{b}_i\right)$ with $-,+$ for each stack $l = 1,2$. The corresponding Dirac cones in each valley $\eta = \pm 1$ of the individual graphene layers are located at $\boldsymbol{K}_\eta^l = -\eta\left(2\boldsymbol{b}_1^l + \boldsymbol{b}_2^l\right)/3$. The emerging moiré pattern [with triangular Bravais lattice shown as green domains in Fig. 1a] is represented by the difference of the new lattice vectors from each bilayer stack in reciprocal space as $\boldsymbol{G}_i^M = \boldsymbol{b}_i^1 - \boldsymbol{b}_i^2$ $(i = 1,2)$, with corresponding primitive lattice vectors $\boldsymbol{L}_j^M$ obtained from the relation $\boldsymbol{G}_i^M \cdot \boldsymbol{L}_j^M = 2\pi\delta_{ij}$.

We consider a description of the low-energy physics for TDBG via the continuum Hamiltonian of Koshino et al..[1] In the Bloch basis given by carbon's p_z orbitals $(A_1, B_1, \ldots, A_4, B_4)$, with sublattices $s = \{A_\ell, B_\ell\}$ and layers $\ell = 1,2,3,4$, the continuum Hamiltonian in valley η for small twist angles $(\theta \ll 1)$ in AB-AB double bilayer graphene can be written as

$$H_{AB-AB} = \begin{pmatrix} H_0(\boldsymbol{k}_1) & S^\dagger(\boldsymbol{k}_1) & & \\ S(\boldsymbol{k}_1) & H_0'(\boldsymbol{k}_1) & U^\dagger & \\ & U & H_0(\boldsymbol{k}_2) & S^\dagger(\boldsymbol{k}_2) \\ & & S(\boldsymbol{k}_2) & H_0(\boldsymbol{k}_2') \end{pmatrix} + V \qquad \text{Supplementary Equation 1}$$

with Bloch wavevectors generated by $\boldsymbol{k}_j = R(\mp\theta/2)\left(\boldsymbol{k} - \boldsymbol{K}_\eta^j\right)$ $(j = 1,2)$ and single-layer graphene Hamiltonians with $k_\pm = \eta k_x \pm i k_y$ as

$$H_0(\boldsymbol{k}) = \begin{pmatrix} 0 & -\hbar\nu k_- \\ -\hbar\nu k_+ & d \end{pmatrix} \quad \text{and} \quad H_0'(\boldsymbol{k}) = \begin{pmatrix} d & -\hbar\nu k_- \\ -\hbar\nu k_+ & 0 \end{pmatrix}. \qquad \text{Supplementary Equation 2}$$

The explicit matrix structure in Supplementary Equation 2 refers to sublattice space (associated with Pauli matrices ρ_j in the main text). In turn, H_0 and H_0' are coupled via

$$S(\boldsymbol{k}) = \begin{pmatrix} \hbar\nu_4 k_+ & \gamma_1 \\ \hbar\nu_3 k_- & \hbar\nu_4 k_+ \end{pmatrix}, \qquad \text{Supplementary Equation 3}$$

* joaoaugustosds@gmail.com

with parameters $\{d, \hbar\nu/a, \gamma_1, \nu_3, \nu_4\} = \{0.050, 2.776, 0.4, 0.32, 0.044\}$ eV and $\nu_i = \left(\sqrt{3}/2\right)\gamma_i a/\hbar\,(i = 3, 4)$. For more details about the physical significance of each term, see Koshino et al..[1] Considering a self-consistently calculated screened electric field, the interlayer potential matrix reads as

$$V = \mathrm{diag}\left(\Delta_1\rho_0, \Delta_2\rho_0, \Delta_3\rho_0, \Delta_4\rho_0\right) \qquad \text{Supplementary Equation 4}$$

where ρ_0 is the unit matrix in sublattice space. For a filling fraction of $\nu = 0.475$, on-site potentials representing the electrostatic energy between adjacent layers are given by $\Delta = (\Delta_1, \Delta_2, \Delta_3, \Delta_4) = (4.079, 1.021, -1.537, -3.563)$ meV,[2] which we use in our numerical calculations.

Finally, the moiré interlayer coupling defined between the twisted layers $\ell = 2 - 3$ is given by

$$U = \begin{pmatrix} u & u' \\ u' & u \end{pmatrix} + \begin{pmatrix} u & u'\omega^{-\eta} \\ u'\omega^{\eta} & u \end{pmatrix} e^{i\eta \boldsymbol{G}_1^M \cdot \boldsymbol{r}} + \begin{pmatrix} u & u'\omega^{\eta} \\ u'\omega^{-\eta} & u \end{pmatrix} e^{i\eta\left(\boldsymbol{G}_1^M + \boldsymbol{G}_2^M\right)\cdot \boldsymbol{r}} \qquad \text{Supplementary Equation 5}$$

with $\omega = \exp 2\pi i/3$, $u = 0.0797$ eV and $u' = 0.0975$ eV. These parameters were chosen in accordance with Samajdar et al. and Koshino et al..[1,2]

From the continuum Hamiltonian in Supplementary Equation 1, a numerical diagonalization in momentum space is performed by selecting a finite number of $\boldsymbol{q}$ wavevectors in a cutoff circle $|\boldsymbol{q} - \boldsymbol{q}_0| < q_c$, with radius $q_c = 4|\boldsymbol{G}_i^M|$ around the midpoint $\boldsymbol{q_0} = \left(\boldsymbol{K}_\eta^1 + \boldsymbol{K}_\eta^2\right)/2$ between Dirac cones $\boldsymbol{K}_\eta^j$. Here, the Bloch vector $\mathbf{k}$ in the moiré Brillouin zone is hybridized with the graphene eigenstates at $\boldsymbol{q} = \boldsymbol{k} + \mathbf{G}_{n_1,n_2}$ due to the coupling between Bernal bilayers via Supplementary Equation 5, with $\mathbf{G}_{n_1,n_2} = n_1\mathbf{G}_1^M + n_2\mathbf{G}_2^M\,(n_1, n_2 \in \mathbb{Z})$. Since we do not consider the intervalley graphene nematicity,[2] the calculations are performed with a fixed valley index, e.g., $\eta = +1$. The corresponding band structure for $\eta = -1$ can be obtained by a time-reversal symmetry transformation. For a certain band n, the wave functions, truncated up to a wavevector $\mathbf{G}_{n_1,n_2}^c$ in the reciprocal lattice, are represented as

$$\Psi_n(\mathbf{k}) = \left(\psi_{n,\mathbf{k}}\left(\mathbf{G}_{n_1,n_2}^1\right), \cdots, \psi_{n,\mathbf{k}}\left(\mathbf{G}_{n_1,n_2}^c\right)\right)^T \qquad \text{Supplementary Equation 6}$$

with each term

$$\psi_{n,\mathbf{k}}(\mathbf{G}) = \left(U_{n,\mathbf{k}}^{A_1}(\mathbf{G}), U_{n,\mathbf{k}}^{A_2}(\mathbf{G}), \cdots, U_{n,\mathbf{k}}^{B_4}(\mathbf{G})\right)^T \qquad \text{Supplementary Equation 7}$$

containing elements in layer ℓ and sublattice s spaces. From these wave functions, the LDOS mappings $\mathcal{D}_{\boldsymbol{r}_0}(\omega)$ and $\mathcal{D}_{\omega_0}(\mathbf{r})$ are computed from

$$\begin{aligned}\mathcal{D}(\boldsymbol{r}, \omega) = &\sum_{n,\boldsymbol{k}} \sum_{\boldsymbol{G},\boldsymbol{G}'} e^{-i\left(\boldsymbol{G}-\boldsymbol{G}'\right)\boldsymbol{r}} \delta\left(\omega - \omega_{n,\boldsymbol{k}}\right) \\ &\times \left(\left[U_{n,\boldsymbol{k}}^{A_4}(\boldsymbol{G}')\right]^* U_{n,\boldsymbol{k}}^{A_4}(\boldsymbol{G}) + \left[U_{n,\boldsymbol{k}}^{B_4}(\boldsymbol{G}')\right]^* U_{n,\boldsymbol{k}}^{B_4}(\boldsymbol{G})\right).\end{aligned} \qquad \text{Supplementary Equation 8}$$

where $\omega_{n,\boldsymbol{k}}$ is the corresponding eigenvalue to the wave function $\Psi_n(\boldsymbol{k})$. This is already projected onto the topmost graphene layer $\ell = 4$, where tunneling of electrons from the STM tip are expected to occur in the experimental setup.

To transform the one-dimensional map of LDOS as a funtion of energies $\mathcal{D}_{\boldsymbol{r}_0}(\omega) = \{\mathcal{D}(\omega_0), \cdots, \mathcal{D}(\omega_{N-1})\}_{\boldsymbol{r}_0}$ into image inputs for the CNN, we use continuous wavelet transforms (CWT).[3,4] These are defined as

$$W(t, h) = \frac{1}{\sqrt{s}} \sum_{i=0}^{N_\omega - 1} \mathcal{D}_{\boldsymbol{r}_0}(\omega_i)\psi\left(\frac{i-t}{h}\right), \quad \text{with} \quad \psi(t) = e^{-t^2/2}\cos(5t), \qquad \text{Supplementary Equation 9}$$

representing the mother wavelet function in a real Morlet form. Here, the transformation is linearly spaced, with $h = 1, 2, \ldots 65$ being equivalent to the spacing in energy taken in the maps $\mathcal{D}_{\boldsymbol{r}_0}(\omega)$. This scale factor is analogous to frequency in Fourier transforms. Besides h, there is a time scale t which is also taken as $t = 1, 2, \ldots 65$, such that $W(t, h)$ produces 65×65 pixel images. An example of such a "scaleogram" is shown in Supplementary Fig. 1.

Finally, the LDOS pixel intensities in both maps $\mathcal{D}_{\boldsymbol{r}_0}(\omega)$ and $\mathcal{D}_{\omega_0}(\mathbf{r})$ are modified by the addition of Gaussian random noise via $p_g(z) = \exp(-z^2/2\sigma^2)/\sqrt{2\pi\sigma^2}$ with $\sigma = 0.31$.[5] For $\mathcal{D}_{\boldsymbol{r}_0}(\omega)$ images, the noise must be added before the CWT for physical consistency.

SUPPLEMENTARY NOTE 2: INCLUDING STRAIN WITH FIXED θ_ϵ, AND VARIATIONS OF THE ML ARCHITECTURE

In this section, we discuss the changes in the performance of the ML procedure when the training data set or the ML architecture are modified. First, we tested in all cases of the main text the performance for the predictions of intensities

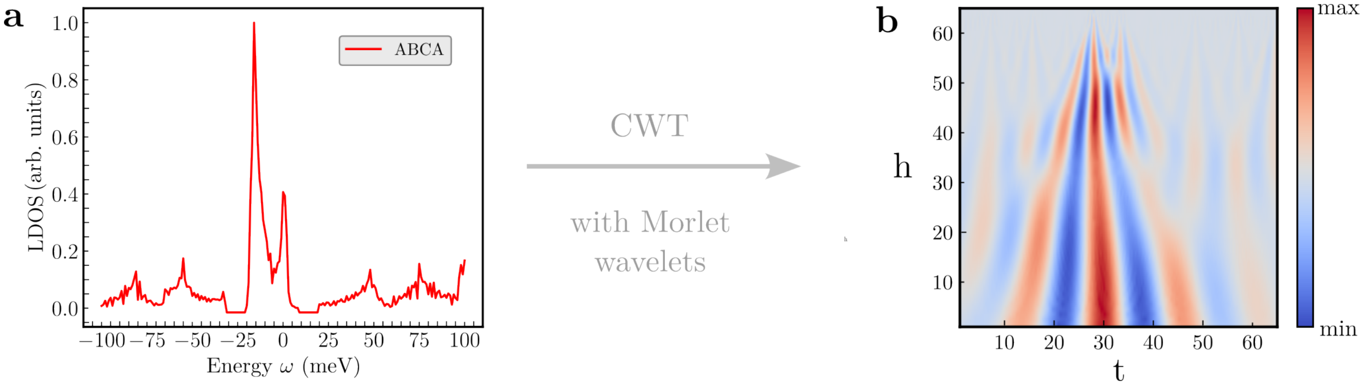

Supplementary Fig. 1 | LDOS $\mathcal{D}_{\mathbf{r}_0}(\omega)$ maps and scaleograms. Local density of states for a fixed position $\mathbf{r}_0 = \text{ABCA}$ as a function of energy ω (**a**) and its corresponding image $W(t, h)$ after a continuous wavelet transform (CWT) (**b**). Intensities are displayed in arbitrary units.

for fixed angles (φ, α and θ_ϵ, respectively). For concreteness, we here focus on predicting the microscopic nematic form in the presence of strain. The data set for this task is, as before, built by randomly sampling nematic and strain intensities $\Phi_{\text{MN}}, \Phi_{\text{GN}} \in [0.001, 0.1]$ eV, and $\epsilon \in [0, 0.8]$ %. Here, $\theta_\epsilon = 0$, $\alpha = 0$, $\psi_l = 1$ and $\varphi_{\text{MN}} = \varphi_{\text{GN}} = \varphi = \pi/3$. In this case, all intensities are easily distinguishable and with high accuracy, see Supplementary Fig. 2. We also repeated this process for 10 different training runs: the maximum of the standard deviation for the predictions of $\beta = \{\Phi_{\text{MN}}, \Phi_{\text{GN}}, \epsilon\}$ is about one to two orders of magnitude smaller than their typical mean value, indicating that the results are robust on different runs. An identical behavior was observed for investigations with fixed φ and α for the predictions of the microscopic form of nematicity, showing that this is a general feature of the considered CNN architecture. Naturally, in the absence of outliers, the precision of the CNN can be further increased (i.e., by reaching a lower MAE for the predictions) with increasing size and variability of the data set.[6]

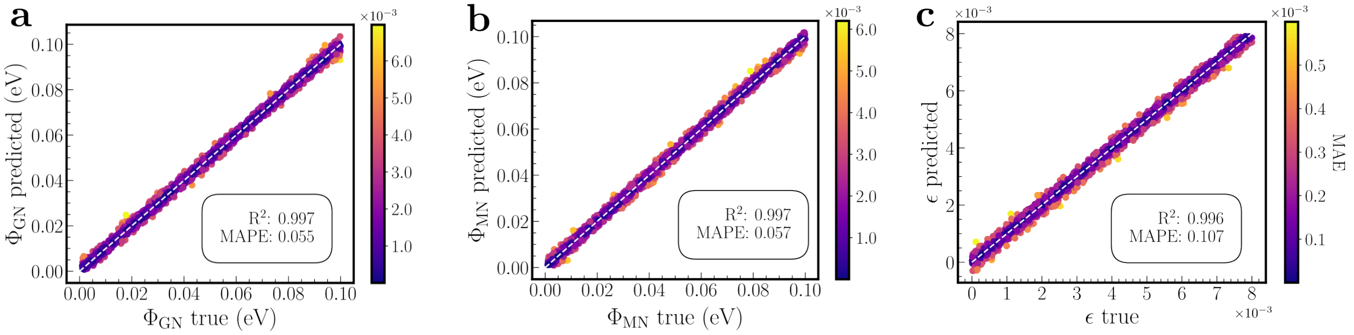

Supplementary Fig. 2 | Predictions with fixed strain angle. Predicted versus true values for graphene nematicity (GN) (**a**), moiré nematicity (MN) (**b**), and strain intensities (**c**) for fixed strain angle $\theta_\epsilon = 0$. Colorbars indicate the mean absolute error (MAE) for each respective case. R squared (R^2) and mean absolute percentage error (MAPE) metrics are also shown in the insets.

We next address whether the complex architecture in Fig. 1a for each channel is really necessary to solve this inverse problem, or in other words, whether simpler architectures could have the same performance and whether modifications of it could produce significant changes in the predictions. For this, we compared the results from the main text with two other architectures in the case of learning the only the microscopic form of nematicity: (i) a very simple sequential neural network that takes the images as inputs, followed by a flatten and dense layer which predicts the parameters $\beta = \{\alpha, \Phi_{\text{GN}}, \Phi_{\text{MN}}\}$; (ii) the architecture in Berthusen et al.,[3] which has a similar structure, i.e., Conv-Batch-MaxPool channel followed by dense layers, but with different number of filters in each layer; we refer to Berthusen et al.[3] for details of the architecture. We have found that, even if there is some clear correlations between the true and the predicted values of α in simple architectures, such as (i), it fails completely on predicting the nematic intensities. Additionally, (ii) does not lead to any significant improvement in the predictions.

We also investigated the performance of the CNN with respect to hyperparameter optimization. These included using different activation functions (SELU, ELU, LeakyReLU, PReLU, ReLU and Sigmoid),[7] batch sizes (9, 16, 32,

48 and 96), different number of filters and convolution layers in the Conv-Batch-MaxPool channels, different learning rates (10^{-1}, 10^{-2}, 10^{-3} and 10^{-4}) and optimizers (RMSprop, SGD and ADAM). The architecture described in Sec. II B and its variation in Fig. 3a already correspond to the optimal configuration. Even though these investigations are not an exhaustive treatment with respect to all possible parameters and correspondent combinations, it shows that certain elements play a major role in the CNN's performance, such as choosing ReLU as activation functions, setting padding to zero in the convolution layers and using a learning rate of 10^{-4}. Finally, even though the four Conv-Batch-MaxPool channels in the main architecture may not be necessary for simpler cases (e.g. predicting the nematic director with fixed nematic intensities), it is essential for increasing parameters and complexity, such as learning strain and the internal structure of nematicity simultaneously.

SUPPLEMENTARY NOTE 3: PREPROCESSING OF THE EXPERIMENTAL DATA AND FURTHER IMPLICATIONS

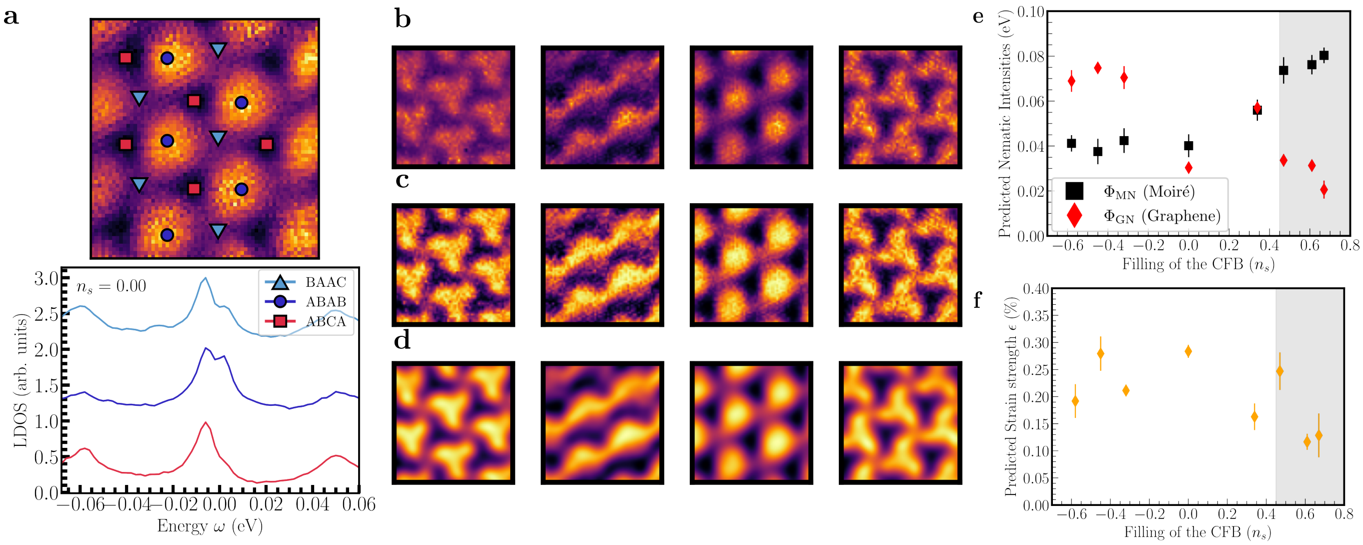

Supplementary Fig. 3 | Preprocessing of experimental data. **a** Location of ABAB, ABCA and BAAC sites in the moiré superlattice for images in $D_{\rm exp}$. The LDOS channels $\mathcal{D}_{\boldsymbol{r}_0}(\omega)$ are built by taking an average over the intensities of equivalent sites for each filling n_s. **b-d** Examples of different preprocessing methods of the $\mathcal{D}_{\omega_0}(\mathbf{r})$ channels: **b** raw data, **c** higher contrast and **d** contrast with Gaussian filter. **e-f** Predictions for the same parameters as in Fig. 5a-b for different preprocessing versions of $\rm D_{exp}$; the markers indicate the average values for predictions on $N = 10$ modified $\rm D_{exp}$ data sets and the error bars represent the corresponding standard deviations, see text for more details.

The experimental data set $D_{\rm exp}(\beta'_1, \cdots, \beta'_{N_{\rm exp}})$ consists of $N_{\rm exp} = 8$ samples for fillings of the CFB equal to $n_s = \{-0.58, -0.45, -0.32, 0, 0.34, 0.47, 0.61, 0.67\}$. The STM measurements were taken at 5K in UHV conditions with a tunnelling set point of 300 mV and 150 pA. Tunneling conductance was measured with a lock-in amplifier with an oscillation amplitude of 1-2 mV. The carrier density was controlled with a voltage applied to a silicon back gate. Each sample has $\mathcal{D}_{\omega_0}(\mathbf{r})$ images in an energy interval of $\omega \in [-100, 100]$ meV with resolution of 2 meV. From these, the $\mathcal{D}_{\mathbf{r}_0}(\omega)$ channels can be calculated by taking an average of intensities at the corresponding BAAC, ABCA and ABAB sites, see Supplementary Fig. 3a.

In order to obtain consistent results, the experimental data set $D_{\rm exp}$ needs to be fed into the trained CNN as similar as possible to the training data in $D_{\rm th}$. For this, the preprocessing of $D_{\rm exp}$ consists of:

(1) Transforming the experimental plots $\mathcal{D}_{\mathbf{r}_0}(\omega)$ into scaleograms as described in Supplementary Fig. 1. Here, these plots are considered for $\omega \in [-70, 60]$ meV. This is necessary in order to have scaleograms of 65×65 pixels. In this energy range, these channels contain information about CFB, VFB, $\rm RV_1$ and $\rm RC_1$.

(2) Normalizing each image to have the distribution of pixel intensities with same mean μ and standard deviation σ in both $D_{\rm exp}$ and $D_{\rm th}$. Here, we have chosen $\mu = 0$ and $\sigma = 1$. This step is essential to produce meaningful predictions on $D_{\rm exp}$, since the trained CNN have weights associated to the scale of $D_{\rm th}$.

(3) Cropping the images $\mathcal{D}_{\omega_0}(\mathbf{r})$ from D_{exp} such that they show roughly the same number of moiré unit cells as in the corresponding ones in D_{th}. Additionally, the orientations of each of these images in both data setss also need to be consistent pair-wisely, see Supplementary Fig. 3b-d and Fig. 5c.

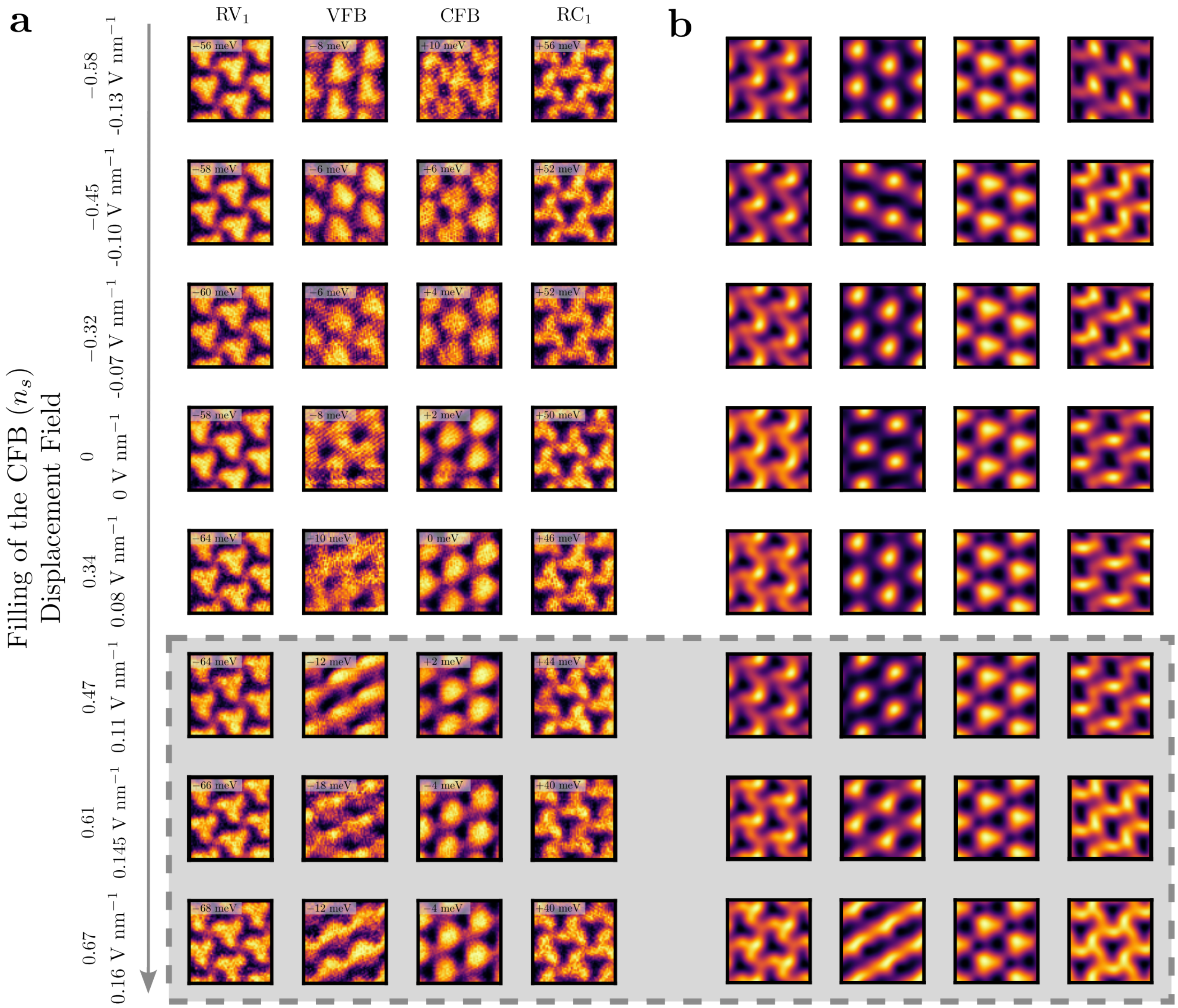

Supplementary Fig. 4 | Predictions for D_{exp} seen from $\mathcal{D}_{\omega_0}(\mathbf{r})$ maps. Comparison between $\mathcal{D}_{\omega_0}(\mathbf{r})$ from the experimental data set D_{exp} (**a**), and the corresponding configuration obtained posteriorly within the continuum model with the predicted β^{exp} (**b**) from Fig. 5a-b. For D_{th} a half-filling fraction of the CFB ($\nu = 0.475$) corresponds to a chemical potential of $\mu \sim -15$ meV, and the equivalent energies for the RV_1, VFB, CFB and RC_1 in the continuum model are found for $\omega_0 = \{-35, -15, 1, 23\}$ meV. These values were chosen for the best possible resemblance of the images $\mathcal{D}_{\omega_0}(\mathbf{r})$ in D_{th} with the ones in D_{exp}, a naturally constrained procedure by the representational power of the theoretical model to the experimental data. The gray box corresponds to the gray regions in Fig. 5a-b. The experimental images are shown with higher contrast (Supplementary Fig. 3c) for better visual comparison.

We have also investigated the influence of additional preprocessing of the $\mathcal{D}_{\omega_0}(\mathbf{r})$ channels of the data set D_{exp} on the predictions. We introduce more contrast to the images (Supplementary Fig. 3c) and reduce noise by smoothing the pixel distribution with a multidimensional Gaussian filter (Supplementary Fig. 3d). In Supplementary Fig. 3e-f, the resulting predictions for the nematicities and strain using these augmented D_{exp} are shown. Here, every dot represents an average over the predictions on 10 variations of D_{exp} with Gaussian filter with standard deviations of the Gaussian kernel in $\sigma_{GF} = \{0, 1, 2, 5, 10\}$ with and without higher contrast. The overall behavior described in

the "Experimental Data" section of the main text is unaffected by these modifications, but the predictions with the lowest strain intensity in the gray region of Fig. 5a-b were found for the raw data inputs (Supplementary Fig. 3b) - see Supplementary Fig. 4.

Finally, we emphasize the importance of the multi-channel CNN architecture in Fig. 3a for the predictions in Fig. 5a-b. While using only $\mathcal{D}_{\omega_0}(\mathbf{r})$ at the flat bands already seems to capture the interplay between MN and GN as a function of fillings of the CFB, the addition of channels for the remote bands and scaleograms is crucial to discern the influence of strain and nematicity in the experimental samples. This can be intuitively understood by the relative stability of the remote bands with respect to heterostrain over a wide range of fillings n_s in D_{exp}.[8] These results indicate that the inclusion of more channels from even more remote bands could potentially produce more accurate predictions for the strain intensity; we leave this for future work.

SUPPLEMENTARY NOTE 4: DETAILS ON TRAINING AND ROBUSTNESS AGAINST PIXEL INHOMOGENEOUS DISORDER IN $\mathcal{D}_{\omega_0}(\mathbf{r})$ MAPS

After creating the data set for the specific task (defined by learning the set of parameters β), we train the models with a mean squared error loss, mean absolute error (MAE) accuracy, ADAM optimizer with a learning rate of 10^{-4}, and a batch size of 64. The training procedure is accompanied by two modules of TensorFlow[7] (i) ModelCheckpoint and (ii) EarlyStopping: the first is responsible for tracking the validation loss and saving the model at the end of each epoch that corresponds to the minimum loss. The EarlyStopping module will interrupt training when it measures no progress on the validation set (recorded by (i)) after a certain number of epochs. This is defined as a patience argument, usually set around $n_{\text{pat}} = 50$. With this, by setting a total number of epochs of 2000, training usually stops around the 400th epoch. A typical loss curve in our trainings can be seen in the blue lines in Supplementary Fig. 5a.

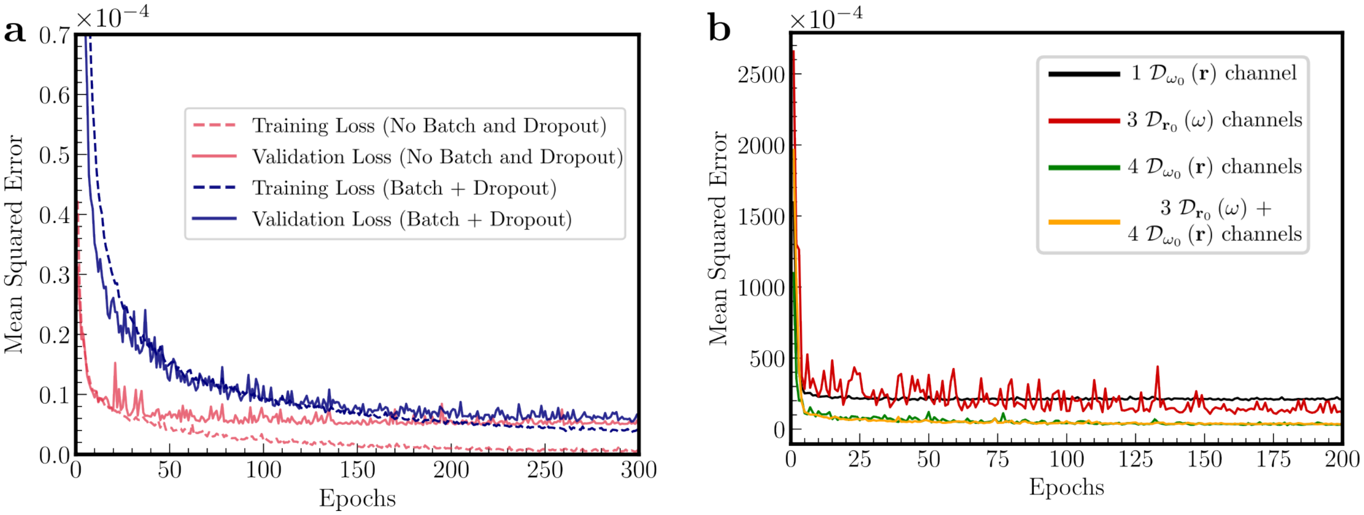

Supplementary Fig. 5 | Training process, overfitting and number of channels. a Loss curves (mean squared error) as a function of the number of epochs for ML architectures with (blue lines) and without (red lines) layers for regularization. The plot was generated with the data set defined by parameters $\beta = \{\Phi_{\text{MN}}, \Phi_{\text{GN}}, \epsilon\}$ from Supplementary Note 2 (see Supplementary Fig. 2 for predictions). **b** Validation loss curves as a function of number of epochs for different combinations of types and numbers of DOS channels for the prediction of $\beta = \{\alpha, \Phi_{\text{MN}}, \Phi_{\text{GN}}\}$.

To exemplify the effect of regularization in the CNN we tested combinations of dropout and batch normalization layers, as well as L1, L2 and L1L2 regularizers for kernel, bias and activation weights on the convolutional layers. Among possible combinations of these regularizes, we have observed a more significant influence on the loss curves by having batch normalization layers before each max pooling layer, and a final dropout layer before the final linear layer for the predictions of parameters β. The dropout ratio is usually set to 20%. From the loss curves in Supplementary Fig. 5a it can be seen that the gap after convergence of losses between validation and training curves is reduced with the addition of regularization layers. As such, our choice of batch normalization and dropout was necessary and effective in avoiding overfitting. All results shown in the main text use this form of normalization. The combination of L1, L2 and L1L2 regularizers for bias did not reduce overfitting as efficiently, and for activation and kernel regularizers in the convolutional layers we actually observed underfitting with a regularization factor of 0.01.

The procedure to select the number of channels in the ML architecture for each section was defined as follows. Naturally, too few channels simply contain not enough information to allow for an accurate prediction of the microscopic parameters (e.g., using only a single $\mathcal{D}_{\omega_0}(\boldsymbol{r})$ was not enough to predict the form of nematicity in Fig. 3 of the main text). While increasing the number of channels of course provides more information and, hence, makes accurate predictions easier in principle, this increases the size of the network and the required (experimental) input data. Importantly, the "optimal" number of channels strongly depends on the task and also the error tolerance. For each section in the main text we typically repeated the training process with different number and types of channel to investigate their influence on the accuracy of predictions. To illustrate this, we consider the data set for predictions of parameters $\beta = \{\alpha, \Phi_{\mathrm{MN}}, \Phi_{\mathrm{GN}}\}$. The validation loss curves are shown in Supplementary Fig. 5b. In this case, the performance with 4 $\mathcal{D}_{\omega_0}(\mathbf{r})$ channels does not get surpassed by the addition of 3 $\mathcal{D}_{\mathbf{r}_0}(\omega)$ channels. When distinguishing strain and nematicity we have found that the inclusion of 7 channels is essential for more accurate predictions on both the theoretical and experimental data sets.

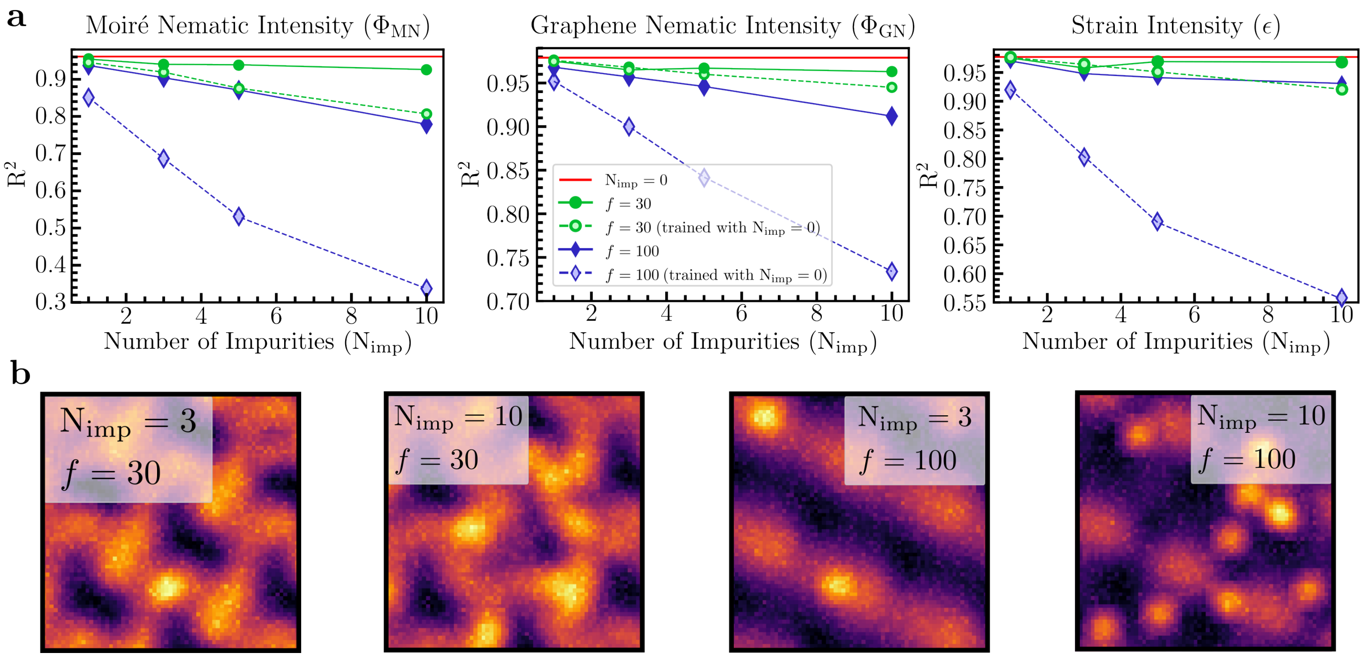

Supplementary Fig. 6 | Learning robustness in the presence of impurities. **a** R^2 metric as a function of increasing number of impurities ($\mathrm{N}_{\mathrm{imp}}$) for predictions of GN, MN and strain intensities. Solid lines represent runs where disorder was also included in the training stage. **b** The effects of this gaussian pixel-disorder are shown in $\mathcal{D}_{\omega_0}(\mathbf{r})$ maps for weaker ($f = 30$) and stronger ($f = 100$) disorder examples.

Finally, in addition to the studies with homogeneous pixel modifications of the test data set in Supplementary Fig. 3b-f, we also investigated the accuracy of the ML approach with the influence of inhomogeneous disorder in the $\mathcal{D}_{\omega_0}(\mathbf{r})$ samples. As such, we consider the same data set from Supplementary Note 2, but now with only 4 $\mathcal{D}_{\omega_0}(\mathbf{r})$ channels. We simulate this on a data set created by the following procedure: randomly sample the position of a certain number of impurities (N_{imp}) which influence locally the LDOS by a Gaussian pixel broadening disorder given by $g(f, \sigma) = f \exp(-r^2/2\sigma^2)/\sqrt{2\pi\sigma^2}$, where f stands for an intensity control. We fix the size of the disorder as 10×10 pixels and $\sigma = 3$. For a given sample (defined by a fixed set of parameters β), the $\mathcal{D}_{\omega_0}(\mathbf{r})$ maps with $\omega_0 = (-35, -15, 1, 23)$ meV have these impurities in the same position for physical consistency. This is repeated for each sample in the entire data set. A few examples can be seen in Supplementary Fig. 6b.

In Supplementary Fig. 6a we show the R^2 metric for the prediction of the parameters $\beta = \{\Phi_{\mathrm{MN}}, \Phi_{\mathrm{GN}}, \epsilon\}$ as a function of increasing number of impurities. The red line represents the case without disorder as a reference point. For weak disorder ($f = 30$), we see that even when impurities are present only on the test data set (dashed green lines) the R^2 metric stays above $\mathrm{R}^2 \geq 0.88$ up to $N_{\mathrm{imp}} = 5$. When including the disorder also on the training data set (solid green lines) we see that the ML procedure seems to learn that the influence of this disorder is not related to the physical parameters β. We also see that predictions on samples with very strong disorder ($f = 100$) become much more accurate if impurities are also considered in the training process (solid blue lines); given the significantly distorted LDOS maps for $f = 100$, $N_{\mathrm{imp}} = 10$, see fourth panel in Supplementary Fig. 6b, the obtained $R^2 \simeq 0.8$ (for moiré nematicity) and $R^2 > 0.9$ (for graphene nematicity and strain) demonstrate the robustness of

the CNN-based methodology against perturbations. These results also suggest that potential instrumental bias can be captured within the ML approach—an important aspect, also for applications beyond nematicity (e.g., concerning the correlated insulator[9,10] or superconductivity) and beyond moiré systems.

SUPPLEMENTARY NOTE 5: APPLICABILITY OF THE CNN TO DIFFERENT MODELS AND MOIRÉ SYSTEMS

To demonstrate that our ML approach of extracting microscopic parameters based on CNNs with multiple channels works more generally, we here apply it to a different moiré system. To further increase the variability of models studied in this work, we do not use a continuum model but, instead, consider a tight-binding model on the moiré scale that captures the symmetries and topological features of the twisted bilayer graphene (TBG).

As is well known,[11–13] the representations of the flat bands of TBG at high-symmetry momenta requires taking a model on the honeycomb lattice. To be able to study the valleys separately, we take the valley quantum number to be conserved such that the associated (fragile) topological obstructions necessitates taking at least four bands.[14] We, therefore, place two Wannier orbitals $W_{\pm}(\boldsymbol{r})$ at every site of the honeycomb lattice. We choose them to be invariant under C_3 (three-fold rotation perpendicular to the graphene layers) and transform into one-another under C_{2x} (two-fold rotation along x) and ΘC_2 (the product of time-reversal and two-fold rotation perpendicular to the layers); this specifies the behavior of the Wannier orbitals under all symmetries of TBG that act within a given valley. Here, our goal is not to provide a quantitatively accurate description of the LDOS of TBG but rather to demonstrate our ML procedure. It is therefore sufficient to take the simple, phenomenological forms of the Wannier states given by

$$W_{\pm}(\boldsymbol{r}) \propto \exp\left(\mp c_1 y\left(y^2-3x^2\right) - c_2\left(x^2+y^2\right)^2\right), \quad \boldsymbol{r}=(x,y)^T, \qquad \text{Supplementary Equation 10}$$

which obey the required symmetry constraints, as shown in Supplementary Fig. 7a. In Supplementary Equation 10, c_1 and c_2 are real-valued constants that we set to $\{c_1, c_2\} = \{1.5, 0.7\}$ for concreteness.

Including symmetry-allowed intra-orbital (inter-orbital) hopping processes up to third-nearest (nearest) neighbor leads to a tight-binding model with momentum-space form (for valley $\eta = +$ and a given spin flavor)

$$\mathcal{H}_{\rm tb} = \sum_{\boldsymbol{k}} c^{\dagger}_{\boldsymbol{k}} h^0_{\boldsymbol{k}} c_{\boldsymbol{k}}, \quad h^0_{\boldsymbol{k}} = \begin{pmatrix} h^{W_+}_{\boldsymbol{k}}(\alpha_2,\Delta) & h^C_{\mathbf{k}} \\ h^{C\dagger}_{\mathbf{k}} & h^{W_-}_{\boldsymbol{k}}(-\alpha_2,-\Delta) \end{pmatrix}, \qquad \text{Supplementary Equation 11}$$

with orbital Hamiltonians

$$h^W_{\boldsymbol{k}}(\alpha_2,\Delta) = \begin{pmatrix} \Delta + f(\alpha_2,t_2) & g(t_1,t_3) \\ g^{\dagger}(t_1,t_3) & -\Delta + f(-\alpha_2,t_2) \end{pmatrix}, \qquad \text{Supplementary Equation 12}$$

coupled via

$$h^C_{\boldsymbol{k}} = \begin{pmatrix} e^{i\Theta\omega}\omega_1 & \omega_2\left(1+2e^{i\frac{\sqrt{3}}{2}k_x}\cos(k_y/2)\right) \\ \omega_2\left(1+2e^{-i\frac{\sqrt{3}}{2}k_x}\cos(k_y/2)\right) & e^{i\Theta\omega}\omega_1 \end{pmatrix}, \qquad \text{Supplementary Equation 13}$$

with

$$f(\alpha_2,t_2) = t_2\left(\cos(\mathbf{k}\cdot\mathbf{a}_2-\alpha_2) + \cos(\mathbf{k}\cdot(\mathbf{a}_1-\mathbf{a}_2)-\alpha_2) + \cos(\mathbf{k}\cdot\mathbf{a}_1+\alpha_2)\right) \qquad \text{Supplementary Equation 14}$$

and

$$g(t_1,t_3) = t_1\left(1+e^{i\mathbf{k}\cdot\mathbf{a}_1}+e^{i\mathbf{k}\cdot\mathbf{a}_2}\right) + + t_3 e^{i\alpha_3}\left(e^{-i\mathbf{k}\cdot(\mathbf{a}_2-\mathbf{a}_1)} + e^{-i\mathbf{k}\cdot(-\mathbf{a}_2-\mathbf{a}_1)} + e^{-i\mathbf{k}\cdot(-\mathbf{a}_2+\mathbf{a}_1)}\right), \qquad \text{Supplementary Equation 15}$$

where $c^{\dagger}_{\boldsymbol{k}}$ are four-component electronic creation operators with the first two (second two) indices referring to the two sublattices of Wannier orbitals W_+ (W_-). Here, the two primitive lattice vectors from monolayer graphene are given by $\mathbf{a}_1/a = \left(\sqrt{3},1\right)/2$ and $\mathbf{a}_2/a = \left(\sqrt{3},-1\right)/2$. In Supplementary Fig. 7b-c, we show the band structure for $\{t_2/t_1, \Delta/t_1, \omega_1/t_1, \omega_2/t_1, \alpha_2, t_3/t_1, \alpha_3, \Theta\omega\} = \{0.6, 0, 0.6, -0.5, 0.3\pi, 0.1, 0.6\pi, 0\}$, which are also used in the ML calculations presented below. We will take the lower two, isolated bands (indicated in black in Supplementary Fig.

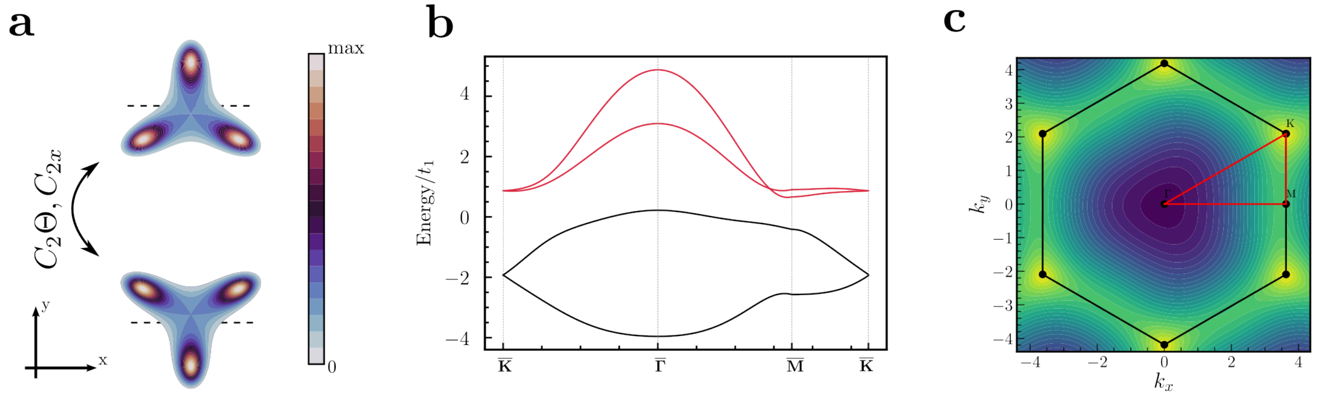

Supplementary Fig. 7 | Minimal model for TBG. a Illustration of the symmetry properties of the Wannier orbitals defined in Supplementary Equation Supplementary Equation 10. In (**b**), we show the bandstructure of the model along the one-dimensional momentum cut indicated in red in (**c**), where the energy of the lowest band is shown as a contour plot. The black bands in (**b**) mimic the flat-bands of TBG, while the red lines are just auxiliary bands required due to the topological obstruction.

7b) as a phenomenological description of the quasi-flat bands of TBG; they exhibit Dirac cones at K and K', which can be shown to have the same chirality—exactly as in TBG.

We next add different forms of nematicity to $h^0_{\boldsymbol{k}}$, i.e., $h^0_{\boldsymbol{k}} \to h_{\boldsymbol{k}} = h^0_{\boldsymbol{k}} + \Delta h_{\boldsymbol{k}}$, which have very different structure compared to those discussed in the main text since the model is very different. The nematic order parameter $\boldsymbol{\phi} = (\phi_1, \phi_2)^T = (\cos 2\varphi, \sin 2\varphi)^T \in \mathbb{R}^2$ will couple as $\Delta h_{\mathbf{k}} = \boldsymbol{\phi} \cdot g_{\mathbf{k}} = \phi_1 g_{1,\mathbf{k}} + \phi_2 g_{2,\mathbf{k}}$. Here, the matrix-valued functions $g_{l,\mathbf{k}}$ play a similar role as the tensorial form factor $\boldsymbol{\phi}_{\sigma,\ell,s,\eta;\,\sigma',\ell',s',\eta'}(\boldsymbol{r}, \Delta\mathbf{r})$ in the continuum model nematic coupling in Equation (1). Denoting by $X_{\boldsymbol{k}}$ and $Y_{\boldsymbol{k}}$ Brillouin-zone-periodic, real-valued functions that transform as x and y under TBG's point group D_3, we can write

$$\mathbf{g}_{\mathbf{k}} = \alpha_0 \rho_0 \sigma_0 \begin{pmatrix} X_{\mathbf{k}} \\ Y_{\mathbf{k}} \end{pmatrix} + \alpha_1 \rho_0 \sigma_x \begin{pmatrix} X_{\mathbf{k}} \\ Y_{\mathbf{k}} \end{pmatrix} + \alpha_2 \rho_0 \sigma_y \begin{pmatrix} -Y_{\mathbf{k}} \\ X_{\mathbf{k}} \end{pmatrix} + \alpha_3 \rho_z \sigma_z \begin{pmatrix} -Y_{\mathbf{k}} \\ X_{\mathbf{k}} \end{pmatrix}, \qquad \text{Supplementary Equation 16}$$

where $\alpha_j \in \mathbb{R}$ are parameters and σ_j (ρ_j) are Pauli matrices in Wannier (sublattice) space. Technically, the explicit form of $X_{\boldsymbol{k}}$ and $Y_{\boldsymbol{k}}$ in each of the four terms in Supplementary Equation Supplementary Equation 16 can be different. However, the functional space of possible $X_{\boldsymbol{k}}$ and $Y_{\boldsymbol{k}}$ is technically infinite dimensional and we will focus only on the leading contribution which then also becomes identical for all four terms in Supplementary Equation Supplementary Equation 16 and reads as

$$(X_{\boldsymbol{k}}, Y_{\boldsymbol{k}}) = \frac{8}{3}\left(\cos k_y - \cos\frac{\sqrt{3}k_x}{2}\cos\frac{k_y}{2}, \sqrt{3}\sin\frac{\sqrt{3}k_x}{2}\sin\frac{k_y}{2}\right). \qquad \text{Supplementary Equation 17}$$

Consequently, there are four parameters, $\beta = \{\alpha_0, \alpha_1, \alpha_2, \alpha_3\}$, describing the microscopic form of nematicity in our model. Our goal will be to reconstruct their values from LDOS images, which we compute via

$$\frac{\mathrm{dI}}{\mathrm{dV}}(\mathbf{r}, \omega) \propto \mathrm{Im}\left[\sum_{j,k,\alpha,\beta} W_{\mathbf{R}_{j\alpha}}(\mathbf{r})\, G^R_{\alpha\beta}(\mathbf{R}_j - \mathbf{R}_k, \omega)\, W^*_{\mathbf{R}_{k\beta}}(\mathbf{r})\right] = \mathcal{D}(\mathbf{r}, \omega) \qquad \text{Supplementary Equation 18}$$

with

$$G^R_{\alpha\beta} = (\mathbf{R} - \mathbf{R}', \omega) = \frac{1}{V}\sum_{\mathbf{k}} e^{i\mathbf{k}(\mathbf{R}-\mathbf{R}')} \lim_{\eta\to 0^+}\left(\frac{1}{\omega - h_{\mathbf{k}} + i\eta}\right)_{\alpha,\beta}. \qquad \text{Supplementary Equation 19}$$

The indices α and β of $W_{\mathbf{R}_{j\alpha}}$ in Supplementary Equation Supplementary Equation 18 correspond to four different realizations of the Wannier functions in each unit cell $\boldsymbol{R}_j$, as each of the two orbitals $W_\pm$ can be placed on each of the two sublattices.

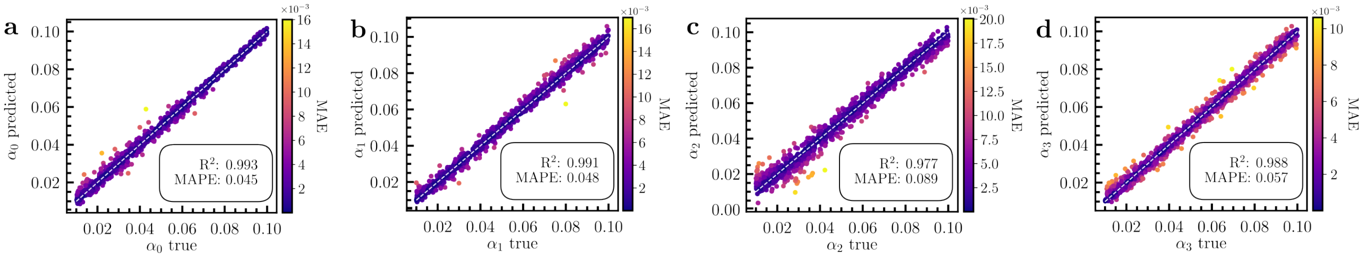

Supplementary Fig. 8 | Predictions for the minimal model. Predicted versus true values for the nematic parameters α_0 (**a**), α_1 (**b**), α_2 (**c**) and α_3 (**d**) defined in Supplementary Equation Supplementary Equation 16 for the minimal model in Supplementary Equation Supplementary Equation 11. As before, colorbars indicate the MAE for each respective case.

To reconstruct $\beta = \{\alpha_0, \alpha_1, \alpha_2, \alpha_3\}$, we consider a variation of the ML architecture in Fig. 3a with four channels for $\mathcal{D}_{\omega_0}(\mathbf{r})$ with $\omega_0/t_1 = \{-2, -1, 1, 2\}$, and one for the scaleograms from $\mathcal{D}_{\mathbf{r}_0}(\omega)$. The complete data set consists of 12000 images which are divided into training (78.5%), validation (15%) and test (6.5%) subgroups. These are generated with randomly sampled $\alpha_j \in [0.01, 0.1]$ for $j = 0, \cdots, 3$ and for a fixed nematic director $\varphi = 5\pi/6$. All the images in the data set were modified by the addition of Gaussian noise with a standard deviation of $\sigma = 0.05$. In Supplementary Fig. 8 a-d, one can see that all four parameters can be accurately predicted. Additionally, we have also observed that even training the CNN with only a single $\mathcal{D}_{\mathbf{r}_0}(\omega)$ channel in this case is sufficient to also yield very good predictions, evidencing the fundamental role of point spectra as an additional source of information. These results indicate that the framework proposed in this work could be successfully applied to a plethora of correlated phenomena and different moiré systems.

Supplementary References

[1] Koshino, M. Band Structure and Topological Properties of Twisted Double Bilayer Graphene. *Phys. Rev. B* **99**, 235406 (2019).

[2] Samajdar, R. et al. Electric-field-tunable electronic nematic order in twisted double-bilayer graphene. *2D Mater.* **8**, 034005 (2021).

[3] Berthusen, N. F., Sizyuk, Y., Scheurer, M. & Orth, P. Learning crystal field parameters using convolutional neural networks. *SciPost Phys.* **11**, 011 (2021).

[4] Mallat, S. A Wavelet Tour of Signal Processing. (Academic Press, San Diego, 1999)

[5] Liu, D., Luskin, M. & Carr, S. Seeing moiré: Convolutional network learning applied to twistronics. *Phys. Rev. Research* **4**, 043224 (2022).

[6] Chollet, F. Deep Learning with Python Second edition (Manning Publications, Shelter Island, 2021).

[7] Abadi, M. et al. TensorFlow: Large-Scale Machine Learning on Heterogeneous Distributed Systems. Preprint at `https://doi.org/10.48550/arXiv.1603.04467` (2016).

[8] Rubio-Verdú, C. et al. Moiré nematic phase in twisted double bilayer graphene. *Nat. Phys.* **18**, 196–202 (2022).

[9] Kim, H. et al. Imaging inter-valley coherent order in magic-angle twisted trilayer graphene. Preprint at `https://doi.org/10.48550/arXiv.2304.10586` (2023).

[10] Nuckolls, K. P. et al. Quantum textures of the many-body wavefunctions in magic-angle graphene. Preprint at `https://doi.org/10.48550/arXiv.2303.00024` (2023).

[11] Kang, J. & Vafek, O. Symmetry, Maximally Localized Wannier States, and a Low-Energy Model for Twisted Bilayer Graphene Narrow Bands. *Phys. Rev. X* **8**, 031088 (2018).

[12] Koshino, M. et al. Maximally Localized Wannier Orbitals and the Extended Hubbard Model for Twisted Bilayer Graphene. *Phys. Rev. X* **8**, 031087 (2018).

[13] Po, H. C., Zou, L., Vishwanath, A. & Senthil, T. Origin of Mott Insulating Behavior and Superconductivity in Twisted Bilayer Graphene. *Phys. Rev. X* **8**, 031089 (2018).

[14] Po, H. C., Zou, L., Senthil, T. & Vishwanath, A. Faithful tight-binding models and fragile topology of magic-angle bilayer graphene. *Phys. Rev. B* **99**, 195455 (2019).
